# Trends in pulmonary exercise testing utilization after the COVID-19 pandemic in Ontario: A population-cohort study

**DOI:** 10.1371/journal.pone.0349020

**Published:** 2026-05-26

**Authors:** Javier Silva-Valencia, Karen Tu, Rahim Moineddin, Debra A. Butt, Braden O’Neill, Anthony Train, Jessica Gronsbell, Andrea S. Gershon

**Affiliations:** 1 Research and Innovation, North York General Hospital, Toronto, Ontario, Canada; 2 Department of Family and Community Medicine, Institute for Health Policy, Management and Evaluation, University of Toronto, Toronto, Ontario, Canada; 3 Toronto Western Family Health Team, University Health Network, Toronto, Canada; 4 Department of Family and Community Medicine, Dalla Lana School of Public Health, University of Toronto, Toronto, Ontario, Canada; 5 Department of Family and Community Medicine, Temerty Faculty of Medicine, University of Toronto, Toronto, Ontario, Canada; 6 BC Psychosis Program, University of British Columbia Hospital, Vancouver Coastal Health, Vancouver, British Columbia, Canada; 7 Department of Family Medicine, Queen’s University, Kingston, Ontario, Canada; 8 Department of Statistical Sciences, University of Toronto, Toronto, Ontario, Canada; 9 Department of Medicine, Sunnybrook Health Sciences Centre, Toronto, Ontario, Canada; Université de Lille: Universite de Lille, FRANCE

## Abstract

**Introduction:**

Pulmonary exercise testing, including six-minute walk tests, exercise oximetry, and independent exercise assessments, are critical tools for managing chronic respiratory and cardiac conditions, evaluating treatment response, and determining long-term oxygen therapy needs. During the COVID-19 pandemic, testing was reduced to limit viral spread. This study aimed to evaluate post-pandemic trends of pulmonary exercise testing utilization in Ontario overall and across demographic groups.

**Methods:**

We conducted a population-based cohort study using Ontario administrative data between April 2015 and December 2023 to evaluate pulmonary exercise testing before, during, and after the COVID-19 pandemic. We used an Auto-Regressive Integrated Moving Average Model (ARIMA) model and incidence rate ratios to evaluate recovery trends. Subgroup analysis examined if trends were similar in different groups.

**Results:**

During the study period, 505,902 tests were performed for 362,888 individuals. As of December 2023, testing rates were still 21% below pre-pandemic levels (IRR 0.79, 95%CI 0.70–0.89). Recovery was lower in males (IRR 0.76, 95%CI 0.66–0.86) and individuals living in lower socioeconomic status neighborhoods (IRR 0.71, 95%CI 0.58–0.86). Northern Ontario saw the most pronounced shortfall compared to other regions, with testing rates one-third of pre-pandemic levels (IRR 0.33, 95% CI 0.26–0.43).

**Conclusion:**

More than three years after the pandemic began, pulmonary exercise testing rates have yet to return to pre-pandemic levels, with certain groups disproportionately affected. This highlights a significant and ongoing disruption in diagnostic capacity and quality of care for people with respiratory and cardiac diseases.

## Introduction

The COVID-19 pandemic significantly disrupted healthcare, resulting in widespread changes to the availability and accessibility of essential medical services. Since the peak of the pandemic, healthcare systems have largely recovered, enabling the return of many health services, including cancer screening, imaging, and surgeries [1–3]. Other essential diagnostic tests took longer. Previously, we reported that over three years after the onset of the pandemic, pulmonary function test rates in Ontario had yet to return to pre-pandemic levels, with certain groups—such as males and individuals of lower socioeconomic status—being disproportionately affected [4].

Pulmonary exercise tests are crucial for evaluating the functional capacity and accompanying oxygenation of individuals with chronic lung and cardiac disease. Tests such as the six-minute walk and independent exercise assessments help document disease progression, evaluate prognosis and response to treatment, determine the need for Long-Term Oxygen Therapy (LTOT), and guide appropriate LTOT oxygen prescriptions [5,6]. These tests are often needed to obtain LTOT from insurers. Lack of testing can result in uncertainty, less precise care, and potential underutilization of LTOT, a lifesaving intervention for many individuals.

There are several mechanisms by which the COVID-19 pandemic disrupted the conduct of pulmonary exercise testing. These tests are typically conducted in pulmonary function laboratories which reduced capacity during the pandemic due to concerns about viral transmission from its aerosol-generating procedures [7], and some temporarily closed. As healthcare services resumed, newly introduced infection control measures aimed at safeguarding patients and healthcare providers made conducting pulmonary exercise testing more resource-intensive. At the same time, demands for pulmonary exercise testing may have increased, as respiratory Long COVID has been shown to be associated with exercise-induced hypoxia, which these tests are used to detect [8,9]. These factors could affect long term available of testing.

To the best of our knowledge, no studies have examined disruptions in pulmonary exercise testing during the pandemic, nor recovery in testing rates after the pandemic. Canadian consensus recommendations from 2021 underscored the importance of resuming pulmonary exercise testing and ensure it availability is not compromised [10].

The purpose of the current study was to explore trends in pulmonary exercise testing in Ontario, Canada, after the pandemic and specifically determine if rates had recovered to pre-pandemic levels. A secondary objective examined if pulmonary exercise testing rates had recovered in individuals of different ages, sex, socioeconomic status, residence, and region.

## Methods

We conducted a population-based cohort study of pulmonary exercise testing utilization from April 2015 to December 2023 in Ontario, Canada, focusing on changes in uptake with the onset of the COVID-19 pandemic and trends over the subsequent three years. Ontario is the most populous province in Canada, with a population exceeding 16 million and universal health care insurance for virtually all residents. Ethics approval was obtained from the North York General Research Ethics Board (#0256).

### Data sources

We used two provincial health administrative datasets that capture information for all Ontario residents covered by OHIP: The Registered Persons Database (RPDB) and the Ontario Health Insurance Plan (OHIP) database [11]. The RPDB captures demographic information, including birth date, sex, postal code, and mortality data. The OHIP database contains details on physician services, including outpatient visits and procedures such as pulmonary exercise testing. Data for this study were accessed through the Ontario Health Data Platform, a Government of Ontario initiative, from November 2023 to July 2024.

### Population

All individuals aged 7 or older, eligible for provincial health care insurance and residing in Ontario between April 1, 2015, and December 31st, 2023, were included to form an open cohort. Individuals entered the cohort when they turn 7 and exited if they moved out of the province or died during the study period. Age 7 or older was chosen because few would dispute that pulmonary exercise testing could be indicated and reliably conducted in children of this age [12].

### Variables

Our primary outcome was the monthly utilization of pulmonary exercise testing, which was assessed in terms of absolute counts and rates of individuals in the population undergoing a test. Records were identified using OHIP billing codes for the 6-minute walk test (J332), exercise oximetry (J334), and independent exercise assessment (J336). Coding for these tests is most likely accurate as they reflect payment for them to be done. There were no changes in coding over the study period, however, using the codes we were not able to assess test quality. Subgroup analyses included additional variables such as age, sex (gender data was not available), rurality, socioeconomic status quintile and asthma and/or COPD status. Rurality and socioeconomic status were determined ecologically by linking postal codes to neighborhood-level data on average income and rurality [13]. The diagnosis and date of asthma and/or COPD were identified using validated algorithms to define patient cohorts [14,15].

### Statistical analysis

#### Descriptive analysis.

Absolute monthly counts of pulmonary exercise testing were determined and categorized by specific test types. Characteristics of individuals who received a test were determined for the years 2019 (pre-pandemic), 2020 (pandemic, with strict public health restrictions), 2022 (early post-pandemic, following the easing of most public health restrictions in Ontario beginning January 2022), and 2023 (late post-pandemic, when nearly all restrictions were lifted). These divisions reflect changes in provincial public health policies and service availability.

#### Pulmonary exercise testing rates.

Monthly pulmonary exercise testing rates were calculated using a dynamic population denominator based on the open cohort. Rates were calculated by dividing the number of individuals receiving at least one pulmonary exercise test in that month by the corresponding population included in that month, expressed per 100,000 persons. We conducted two complementary time-series analyses addressing different study objectives.

First, to assess the impact of the COVID-19 pandemic on test rates, we performed an interrupted time-series analysis adjusted for seasonality and serial correlation to estimate both an abrupt (step change) and a gradual shift over time (trend change), as described elsewhere [16]. Second, to evaluate recovery relative to expected pre-pandemic patterns, we developed a separate seasonal ARIMA forecasting model using data from April 2015 to February 2020. This model generated counterfactual expected rates from March 2020 to December 2023 under a no-pandemic scenario. Incidence rate ratios (IRRs) with 95% confidence intervals were calculated by comparing observed post-pandemic rates to these expected values.

Model fit was assessed by examining residuals using autocorrelation and partial autocorrelation functions, as well as normality tests. Subgroup analyses were conducted using the same modelling framework. All analyses were performed in R version 4.3.2.

## Results

### Pulmonary exercise testing trends

**Fig 1:**
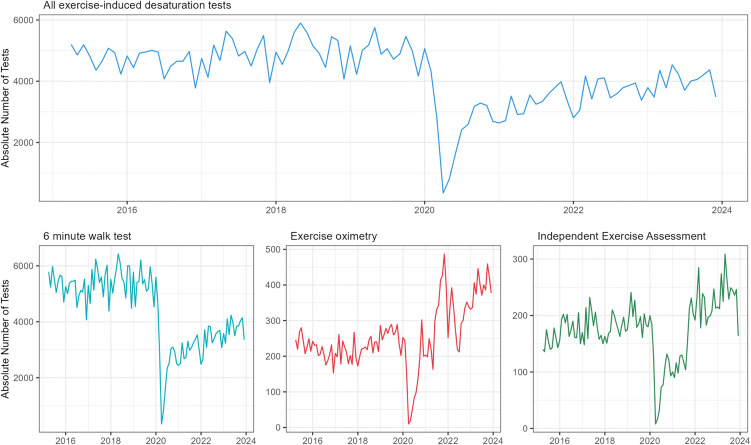
All exercise tests.

There were 505,902 pulmonary exercise tests performed in Ontario during the study period (2015–2023). Of these, 91.0% were 6-minute walk tests, 5.3% were exercise oximetry tests, and 3.7% were independent exercise assessments. This distribution remained relatively consistent across study years (S1 Appendix). The monthly pre-pandemic average was 5,730 (±633) tests. In April 2020, this number decreased to 366 tests, representing a drop of approximately 94%. (Fig 1) After this decline, test volume began to recover. By 2023, the average monthly count from January to December reached 4,415(±378) tests. In later months, exercise oximetry and independent exercise assessments surpassed pre-pandemic levels, likely to address built-up demand. Six-minute walk test volumes remained below pre-pandemic levels through the end of the study.

### Persons receiving an exercise test

Among the eligible population aged 7 or older of approximately 12.4 million, 362,888 persons received at least one exercise test during the study period. Characteristics of people who received testing remained similar throughout the years. The mean age of tested individuals was 58 (±17) years, with males and females equally represented. Most tests (83%) were performed in urban areas, with the largest proportion of tests conducted in Ontario’s West and East regions. About half of people who received pulmonary exercise testing had asthma and/or COPD. (Table 1)

**Table 1:** Characteristics of persons who received pulmonary exercise testing during representative study years.

Characteristic	Year 2019 (before pandemic) n = 48,088	Year 2020 (pandemic year) n = 28,708	Year 2022 (early post-pandemic) n = 37,030	Year 2023 (late post-pandemic) n = 40,083
Sex, n (%)				
Female	24,130 (50.2%)	14,339 (49.9%)	18,876 (51.0%)	20,497 (51.1%)
Male	23,958 (49.8%)	14,369 (50.1%)	18,154 (49.0%)	19,586 (48.9%)
Age at time of test, years				
Mean (SD)	58(17)	56(17)	56(17)	55(17)
Median (IQR)	61(21)	59(21)	59(21)	58(21)
Age group, n (%)				
Less than 18 years	1,141 (2.4%)	711 (2.5%)	948 (2.6%)	1,155 (2.9%)
18 to 64 years	22,437 (46.7%)	13,591 (47.3%)	16,126 (43.5%)	17,039 (42.5%)
More than 65 years	24,510 (51.0%)	14,406 (50.2%)	19,956 (53.9%)	21,889 (54.6%)
Ontario region, n (%)				
Central	7,913 (16.5%)	5,118 (17.8%)	6,982 (18.9%)	7,450 (18.6%)
East	16,766 (34.9%)	9,482 (33.0%)	11,619 (31.4%)	12,735 (31.8%)
North (East & West)	5,662 (11.8%)	2,534 (8.9%)	1,771 (4.8%)	1,931 (4.8%)
Toronto	5,818 (12.1%)	3,422 (11.9%)	5,109 (13.8%)	5,692 (14.2%)
West	11,897 (24.7%)	8,112 (28.3%)	11,530 (31.1%)	12,254 (30.6%)
Unknown	32 (0.1%)	13 (0.0%)	19 (0.1%)	21 (0.1%)
Rurality, n (%)				
Rural	8,352 (17.4%)	5,033 (17.5%)	6,377 (17.2%)	6,801 (17.0%)
Urban	39,736 (82.6%)	23,675 (82.5%)	30,653 (82.8%)	33,282 (83.0%)
Income Quintile, n (%)				
Q1 - Lowest	11,440 (23.8%)	6,914 (24.1%)	8,532 (23.1%)	9,041 (22.6%)
Q2	9,932 (20.7%)	5,890 (20.5%)	7,461 (20.2%)	8,075 (20.2%)
Q3 - Middle	8,940 (18.6%)	5,293 (18.5%)	7,110 (19.2%)	7,611 (19.0%)
Q4	8,708 (18.1%)	5,256 (18.3%)	6,810 (18.4%)	7,528 (18.8%)
Q5 - Highest	9,011 (18.8%)	5,316 (18.5%)	7,069 (19.1%)	7,782 (19.4%)
Unknown	57	39	48	46
Chromic Respiratory Condition, n (%)				
Only Asthma	5,023 (10.4%)	3,123 (10.9%)	4,670 (12.6%)	4,854 (12.1%)
Asthma & COPD	5,255 (10.9%)	3,126 (10.9%)	4,380 (11.8%)	4,606 (11.5%)
Only COPD	12,932 (26.9%)	7,805 (27.2%)	9,817 (26.5%)	10,498 (26.2%)
None	24,878 (51.7%)	14,654 (51.0%)	18,163 (49.0%)	20,125 (50.2%)

### Pulmonary exercise testing rates

**Fig 2:**
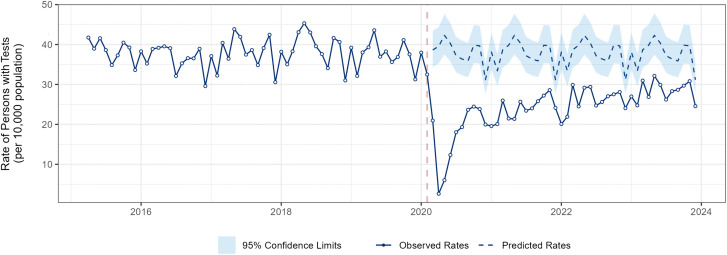
Interrupted time-series graph showing the impact of the COVID-19 pandemic on monthly rates of persons undergoing pulmonary exercise testing in Ontario. The solid blue line represents the observed rates. The dashed blue line represents the expected rates if no pandemic exists. The 95% confidence limit of the expected rates is shown in blue shading.

Before the pandemic, an average of 37.9 people received pulmonary exercise testing per 100,000 individuals. (Fig 2) At the onset of the pandemic, test rates dropped by 56%, corresponding a decline of 21.3 (95%CI −24.62 to −18.05) people per 100,000 individuals. This decline was observed across all demographic groups. (Table 2) The largest initial reductions occurred among individuals aged 85 years and older (60% decline) and residents of Ontario’s northern region (75% decline), relative to their respective pre-pandemic rates.

**Table 2:** Results of interrupted time series analyses of the impact of the COVID-19 pandemic on the rate of persons with pulmonary exercise testing.

Characteristics	N	Average monthly rate prior pandemic (April 2015 to February 2020)	Drop in Rates of test uptake at the beginning of COVID-19 Pandemic* Change in rate (95% confidence interval)	Average monthly recovery trend following the pandemic onset Change in rate (95% confidence interval)
All persons	441,700	37.93	−21.33 (−24.62 to −18.05)	+0.31 (0.2 to 0.43)
Sex				
Female	220,098	38.80	−21.27 (−24.62 to −17.91)	+0.28 (0.17 to 0.4)
Male	221,602	37.05	−21.27 (−24.6 to −17.94)	+0.34 (0.22 to 0.46)
Age group				
18-34	24,485	8.67	−4.39 (−5.23 to −3.54)	+0.06 (0.03 to 0.09)
35-64	178,121	35.78	−20.35 (−23.78 to −16.92)	+0.23 (0.11 to 0.36)
65-74	121,239	99.67	−56.9 (−66.47 to −47.34)	+0.72 (0.38 to 1.07)
75-84	84,932	127.28	−70.99 (−83.29 to −58.7)	+1.02 (0.57 to 1.47)
85+	23,858	80.96	−48.62 (−57.87 to −39.38)	+0.98 (0.66 to 1.29)
Rurality				
Urban	71,322	41.27	−21.49 (−26.29 to −16.69)	+0.38 (0.22 to 0.54)
Rural	369,512	37.28	−21.04 (−24.22 to −17.87)	+0.29 (0.18 to 0.41)
Income Quintile				
Q1-Lowest	106,469	43.67	−24.6 (−28.33 to −20.86)	+0.29 (0.16 to 0.43)
Q2	92,003	39.89	−22.97 (−26.4 to −19.54)	+0.31 (0.19 to 0.43)
Q3-Middle	82,262	35.90	−19.78 (−23.1 to −16.47)	+0.29 (0.17 to 0.41)
Q4	79,163	35.44	−20.63 (−24.14 to −17.12)	+0.34 (0.22 to 0.47)
Q5-Highest	81,221	34.22	−19.42 (−22.73 to −16.11)	+0.35 (0.24 to 0.47)
Ontario region				
West	73,160	24.00	−12.32 (−15.65 to −9)	+0.22 (0.1 to 0.34)
Toronto	149,068	62.17	−36.68 (−42.11 to −31.24)	+0.49 (0.3 to 0.68)
North	39,815	71.14	−53.37 (−61.53 to −45.22)	+0.16 (−0.11 to 0.44)
East	60,623	26.64	−14.79 (−19.16 to −10.42)	+0.24 (0.06 to 0.42)
Central	116,629	32.24	−15.06 (−18.09 to −12.03)	+0.35 (0.24 to 0.45)

Rates represent the number of individuals undergoing pulmonary function testing per 100,000 population (95% confidence interval).

Negative values indicate a decrease and positive values indicate an increase. * Represent the drop in rates in the first month of the pandemic

Following this initial drop, testing rates started to recover across most groups. However, recovery patterns varied. The most pronounced rebounds were observed in individuals aged 75–84 and 85 + years. In contrast, there was no significant evidence of recovery in northern Ontario.

### Recovery after pandemic onset

**Fig 3:**
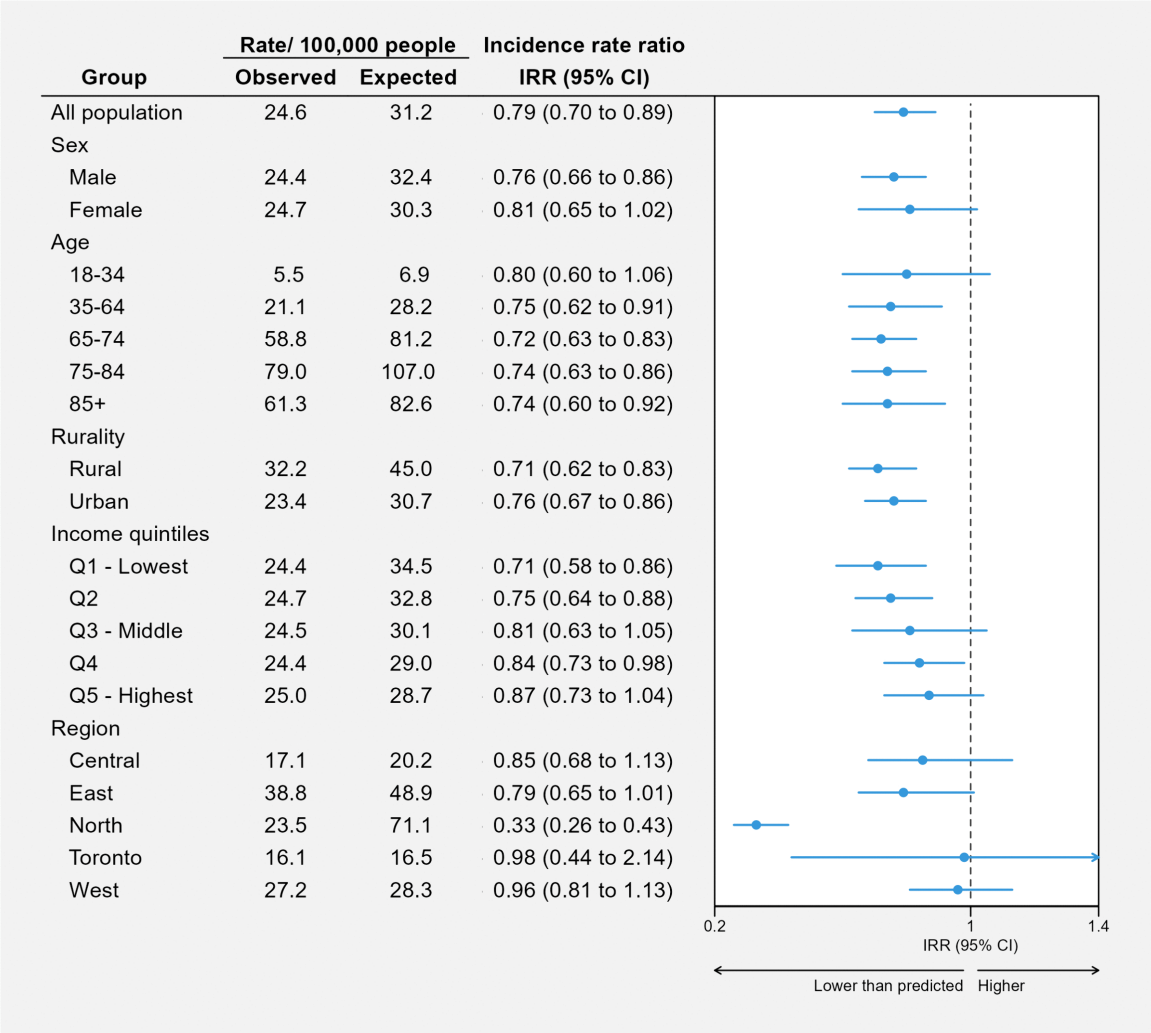
Rates, absolute rate differences, and incidence rate ratios of persons receiving pulmonary exercise testing in Ontario per 100,000 population in December of 2023, comparing observed with expected rates if the pandemic had not occurred.

As of December 2023, pulmonary exercise testing rates had not recovered to pre-pandemic levels. Fig 3 compares observed rates of pulmonary exercise testing with rates that would be expected had the pandemic not occurred across various groups. While no group fully recovered, the shortfall was more pronounced in certain populations. Testing rates remained 24% lower among men (IRR 0.76, 95% CI 0.66–0.86), 29% lower in those of lower socioeconomic status quintile (IRR of the lowest socioeconomic status quintile 0.71, 95% CI 0.58–0.86) and 67% lower in people living in northern Ontario (IRR 0.33, 95% CI 0.26–0.43).

## Discussion

We conducted a population-based cohort study of pulmonary exercise testing in a large Canadian population and found prolonged disruption in pulmonary exercise testing rates that persisted three years after the onset of the COVID-19 pandemic. This was most pronounced in males, those of lower socioeconomic status, and those living in northern Ontario. People of all ages, as well as those living in both urban and rural areas, were impacted. This indicates a significant and ongoing disruption in the quality of care for individuals with respiratory and cardiac diseases in the post-pandemic period, particularly among certain groups.

To our knowledge, no prior studies have evaluated long-term, population-level recovery trends in pulmonary exercise testing. The limited data available, such as a U.S. study on patients within specific healthcare institutions, suggest that cardiopulmonary exercise and 6-minute walk tests quickly returned to pre-pandemic levels within a few months [17]. Research on other diagnostic services, including cardiac, imaging, and cancer diagnostic testing, indicates that most recovered to pre-pandemic levels within the first year of the pandemic [3,18,19]. In contrast, our findings from Ontario suggest that pulmonary exercise testing, similar to pulmonary function tests [4] had still not fully recovered three years after the pandemic started.

Several factors likely contributed to the persistent reduction in pulmonary exercise testing observed. Many offices and laboratories were temporarily or permanently closed during the pandemic, and more rigorous infection control practices may have further reduced their capacity to perform these tests [10]. A high mortality rate among those who need pulmonary exercise testing during the pandemic, specifically older people and people with advanced respiratory disease, may have left fewer people who needed such testing after the pandemic reducing the need and rate of testing. These vulnerable individuals may have died during the pandemic from COVID-19 or from not getting proper health care for their comorbidities [20,21].

Pulmonary exercise testing is crucial for assessing exertional hypoxia, a key indicator of conditions like COPD or pulmonary hypertension. Delayed access can lead to inadequate management and delay patients from qualifying for life-saving interventions like LTOT, which reduces mortality in COPD and likely benefits other advanced respiratory and cardiac conditions [6,22]. During the pandemic, the Ontario Ministry of Health temporarily waived the requirement of exercise test results to qualify for LTOT, allowing access without formal assessment. Later, in September 2021, the need for exercise assessment results for LTOT was reinstated [23]. This policy change likely explains the observed increase in the number of exercise oximetry and independent exercise assessment after this time.

While some home-based pulmonary exercise testing gained popularity during the pandemic as an alternative to in-clinic testing, these tests often vary in reliability outside of the controlled laboratory setting. Therefore, they are not a complete substitute for traditional testing [24,25]. While availability of pulmonary exercise testing decreased, relative demand for testing may have increased because of the emergency of Long COVID, a new disease that can cause exertional hypoxia despite normal resting oxygen levels [8,9]. Testing for patients suspected of having this condition would be indicated to document hypoxia and to support personalized rehabilitation plans and to monitor recovery.

Disparities in testing utilization are particularly concerning. We observed that males and individuals from lower socioeconomic groups had lower access to pulmonary exercise testing compared to other Ontarians. It is known that these groups could often face a combination of challenges. Differences by sex may reflect complex factors, including potential differences in healthcare-seeking behaviour, perceived needs, or access and referral pathways, especially in preventive and outpatient settings [26]. Less recovery by December 2023 in rural and northern Ontario compared to other regions is consistent with ongoing challenges in accessing primary healthcare and may reflect workforce shortages, facility closures and limited pulmonary lab capacity to begin with. Geographic barriers and longer travel distances of the northern areas may have further restricted access, particularly for individuals with chronic respiratory or cardiac conditions [27]. These findings highlight the need for further research to confirm these findings, determine the reasons for them and, if appropriate, devise policies to ensure equitable access by underserved groups who may be at risk of being overlooked as healthcare systems recover. Addressing the backlog in pulmonary exercise testing is essential to avoid exacerbating healthcare burdens associated with post-COVID conditions and to support these patients’ health.

This study’s strengths include its large population-based design, its analysis across multiple demographic groups, and the fact that the duration of the study spans almost three and a half years since the onset of the pandemic. However, it also has some limitations. First, we could not determine whether individuals’ needs were met, for example, if everyone who needed an exercise test during the pandemic received one in a timely manner. Additionally, it remains unclear whether testing capacity addressed cumulative demand, particularly if a backlog of unmet needs persisted after years of reduced testing. Second, our analyses relied on administrative health data and physician billing codes. Although these codes are generally considered reliable because they are linked to reimbursement, some minor degree of coding error or misclassification is still possible. While we were able to identify if a test was done, we could not assess its quality, which could also influence clinical decisions and outcomes. Third, we were unable to account for alternative diagnostic methods, such as home-based pulmonary exercise testing, which became more common during the pandemic and may have partially offset demand for traditional testing. Finally, we were unable to measure the full spectrum of patient outcomes related to delayed or foregone testing, particularly among high-risk groups who may have been disproportionately affected.

## Conclusion

We conducted a population study of pulmonary exercise testing in Ontario, Canada and found that more than three years after the pandemic began, rates had not yet returned to pre-pandemic levels, with certain groups being more affected than others. This undermines efforts to deliver quality and potentially life-saving care for individuals with cardiac and respiratory conditions. There is a clear need for advocacy and education to increase testing capacity, especially for high-risk groups. Future research should assess the long-term consequences of diagnostic delays on patient outcomes and identify strategies to restore equitable access, particularly for those at risk of not receiving testing, to guide public health policies and support pandemic recovery efforts.

## Supporting information

S1 Appendix  Number of Pulmonary exercise testing by type performed by year (2015–2023). (PDF)
