# Supplementary material for: Trends in pulmonary exercise testing utilization after the COVID-19 pandemic in Ontario: A population-cohort study
Source: PLoS One. 2026 May 26;21(5):e0349020. doi: 10.1371/journal.pone.0349020 (PMC13210312; doi:10.1371/journal.pone.0349020)
Supplement: S1 Appendix — (PDF) [file pone.0349020.s001.pdf]

S1 Appendix

Number of Pulmonary exercise testing by type performed by year (2015-2023)

Table A. Number of Pulmonary exercise testing by type performed by year (2015-2023)

	2015 (N=52536)	2016 (N=65884)	2017 (N=69815)	2018 (N=71579)	2019 (N=69847)	2020 (N=35185)	2021 (N=41558)	2022 (N=46513)	2023 (N=52985)
**Pulmonary exercise testing**									
6-minute walk test	49,012 (93.3%)	61,241 (93.0%)	65,097 (93.2%)	66,775 (93.3%)	64,357 (92.1%)	32,272 (91.7%)	36,141 (87.0%)	40,265 (86.6%)	45,404 (85.7%)
Exercise oximetry	2,153 (4.1%)	2,498 (3.8%)	2,547 (3.6%)	2,661 (3.7%)	3,156 (4.5%)	1,741 (4.9%)	3,732 (9.0%)	3,642 (7.8%)	4,713 (8.9%)
Independent Exercise Assessment	1,371 (2.6%)	2,145 (3.3%)	2,171 (3.1%)	2,143 (3.0%)	2,334 (3.3%)	1,172 (3.3%)	1,685 (4.1%)	2,606 (5.6%)	2,868 (5.4%)
